# Antithrombotic therapy in antiphospholipid syndrome with arterial thrombosis: a systematic review and network meta-analysis

**DOI:** 10.3389/fmed.2023.1196800

**Published:** 2023-06-15

**Authors:** Tanawat Attachaipanich, Aimpat Aungsusiripong, Pokpong Piriyakhuntorn, Sasinee Hantrakool, Ekarat Rattarittamrong, Thanawat Rattanathammethee, Adisak Tantiworawit, Lalita Norasetthada, Chatree Chai-Adisaksopha

**Affiliations:** ^1^Cardiac Electrophysiology Research and Training Center, Faculty of Medicine, Chiang Mai University, Chiang Mai, Thailand; ^2^Department of Otorhinolaryngology, Faculty of Medicine, Siriraj Hospital, Mahidol University, Bangkok, Thailand; ^3^Division of Hematology, Department of Internal Medicine, Chiang Mai University, Chiang Mai, Thailand

**Keywords:** anticoagulant, antiphospholipid syndrome, network meta-analysis, platelet aggregation inhibitors, thrombosis

## Abstract

**Introduction:**

The optimal secondary thromboprophylactic strategies for patients with antiphospholipid syndrome (APS) and arterial thrombosis remain controversial. This study aimed to evaluate the comparative efficacy and safety of various antithrombotic strategies in APS with arterial thrombosis.

**Methods:**

A comprehensive literature search was conducted using OVID MEDLINE, EMBASE, Web of Science, and the Cochrane Controlled Register of Trials (CENTRAL) from inception until 30 September 2022, with no language restrictions. The inclusion criteria for eligible studies were as follows: inclusion of APS patients with arterial thrombosis, treatment with either antiplatelet agents, warfarin, direct oral anticoagulants (DOACs), or a combination of these therapies, and reporting of recurrent thrombotic events.

**Results:**

We conducted a frequentist random-effects network meta-analysis (NMA) involving 13 studies with a total of 719 participants, comprising six randomized and seven non-randomized studies. In comparison to single antiplatelet therapy (SAPT), the combined use of antiplatelet and warfarin demonstrated a significant reduction in the risk of recurrent overall thrombosis, with a risk ratio (RR) of 0.41 (95% CI 0.20 to 0.85). Dual antiplatelet therapy (DAPT) showed a lower risk of recurrent arterial thrombosis compared to SAPT although the difference did not reach statistical significance, with an RR of 0.29 (95% CI 0.08 to 1.07). DOAC was associated with a significant increase in the risk of recurrent arterial thrombosis, with an RR of 4.06 (95% CI 1.33 to 12.40) when compared to SAPT. There was no significant difference in major bleeding among various antithrombotic strategies.

**Discussion:**

Based on this NMA, the combination of warfarin and antiplatelet therapy appears to be an effective approach in preventing recurrent overall thrombosis in APS patients with a history of arterial thrombosis. While DAPT may also show promise in preventing recurrent arterial thrombosis, further studies are needed to confirm its efficacy. Conversely, the use of DOACs was found to significantly increase the risk of recurrent arterial thrombosis.

## 1. Introduction

Antiphospholipid syndrome (APS) is an autoimmune disease characterized by pregnancy morbidities or thrombotic events, including arterial, venous, or microvascular thrombosis, in the presence of persistent elevated antiphospholipid antibodies (aPL) (1, 2). While venous thrombosis is the most common thrombotic manifestation of APS, arterial thrombosis is also a common occurrence and often has more severe consequences (3). According to the findings from an observational study involving 1,000 patients with antiphospholipid syndrome (APS), it was observed that 19.8% presented with stroke, 11.1% with a transient ischemic attack (TIA), and 5.5% with myocardial infarction (MI) as their initial manifestations (3). Similarly, in a multicenter international registry, it was found that among patients with APS, 37% presented with arterial thrombosis. Specifically, 26% presented with stroke, 11% with TIA, and 5% with MI (4). Despite current treatments, after 10-year follow-ups, the mortality rate in individuals with APS was found to be 9.3%. Notably, severe thrombotic events were responsible for 36.5% of the overall deaths (3).

In the context of secondary thromboprophylaxis for APS with arterial thrombosis, the current recommendation suggests the use of antithrombotic strategies such as high-intensity warfarin with a target international normalized ratio (INR) of 3.0–4.0 or moderate-intensity warfarin with a target INR of 2.0–3.0. Additionally, a combination of moderate-intensity warfarin and aspirin may also be considered a treatment option. These strategies aim to prevent further thrombotic events in individuals with APS and arterial thrombosis (5). However, there is still a lack of consensus on optimal antithrombotic regimens in this context (5, 6). Therefore, we conducted a systematic review and network meta-analysis (NMA) to assess the efficacy and safety of antithrombotic strategies in this population.

## 2. Methods

### 2.1. Search strategy, selection criteria, and data extraction

A systematic review and NMA were conducted with a standard outline of the preferred reporting items for systematic reviews and meta-analyses (PRISMA) (7). We searched the data from four databases, namely OVID MEDLINE, Web of Science, EMBASE, and the Cochrane Central Register of Controlled Trials (CENTRAL), from inception to a final search date of 30 September 2022, with no language restriction. We applied search terms related to APS, aPL, arterial thrombosis, venous thrombosis, antiplatelet, anticoagulant, and direct oral anticoagulants (DOACs). The full search strategies are provided in the Supplementary material.

The inclusion criteria for studies in this systematic review and network meta-analysis (NMA) were as follows: (1) studies that involved patients diagnosed with APS who initially presented with arterial thrombosis, (2) treatment with at least one antithrombotic regimen for secondary thromboprophylaxis, including antiplatelets, anticoagulants, DOACs, or combination of these treatments, (3) reporting of recurrent thrombotic events as a study endpoint (either arterial or venous thrombosis), and (4) inclusion of randomized or observational studies. Studies were excluded from the analysis if they met any of the following criteria: (1) failure to demonstrate the persistently elevated aPL according to APS definition, (2) inclusion of case series, case reports, or small studies with <5 participants in each treatment arm, or (3) absence of separate reporting of thrombotic outcomes specifically in APS patients with arterial thrombosis, distinct from other presentations. We included both full articles and conference abstracts that fulfilled the abovementioned criteria. We used the Sapporo or revised Sapporo classification as diagnostic criteria for APS diagnosis.

Two investigators (TA and AA) independently reviewed abstracts and full texts to select the eligible studies. Discrepancies were resolved through discussion and reviewed by three reviewers (TA, AA, and CC). The final consensus was made with the agreement of the three reviewers. The primary efficacy outcome was the composite of recurrent arterial and venous thrombosis. The primary safety outcome was major bleeding which was based on the definition in each study protocol. Secondary outcomes were recurrent arterial thrombosis, venous thrombosis, and all-cause mortality.

The data extraction from eligible studies was performed independently by two investigators (TA and AA), using a standardized data extraction sheet. The extracted data included study design, baseline characteristics, initial arterial thrombotic presentation, APS classification criteria, intervention, comparator, and outcomes. For the analysis, only the events specifically related to the subgroup of APS patients with previous arterial thrombosis were extracted from each therapeutic arm. This approach ensures that the analysis focuses specifically on the outcomes and effectiveness of the treatments in APS patients with a history of arterial thrombosis, allowing for a more targeted and informative assessment. Corresponding authors were contacted to obtain unpublished or unclarified data. In cases where multiple studies reported the same or overlapping participants, only the study with the largest sample size was included in the analysis. The methodological quality of the randomized studies was evaluated independently by two reviewers using a revised Cochrane risk-of-bias tool for randomized trials (RoB 2) (8). The quality of a non-randomized study was evaluated by using a Risk Of Bias In Non-randomized Studies of Interventions (ROBINS-I) (9).

### 2.2. The geometry of the network and summary measures

We conducted an NMA using a frequentist random-effects model. Six nodes represented each antithrombotic strategy, including single antiplatelet therapy (SAPT), dual antiplatelet therapy (DAPT), DOACs, high-intensity warfarin, moderate-intensity warfarin, and combined warfarin and antiplatelet. In this analysis, we made the assumption that all DOACs, including rivaroxaban, edoxaban, apixaban, and dabigatran, had comparable efficacy. As a result, these medications were grouped together as DOACs in the analysis. The risk ratio (RR) and 95% confidence interval (CI) were estimated by comparing each antithrombotic regimen to SAPT as a reference. The effect size was represented in the form of a forest plot. To rank the best antithrombotic strategies, a P-score was calculated by using the net rank function. The P-score reflected the certainty of one treatment being better than other treatments, which was shown to be equivalent to a Surface under the Cumulative Ranking (SUCRA) score (10).

The values of I^2^ and Cochran's Q, which represented the inconsistency and heterogeneity in the network, were calculated (11). The net heat plot and net-splitting approach were performed to evaluate the inconsistency between direct and indirect comparisons in an NMA. The publication bias was assessed by using a comparison-adjusted funnel plot. All results were analyzed using the netmeta package in R, version 3.6.2. (12, 13). A *p* < 0.05 was considered to be statistically significant.

A GRADE approach was applied to assess the certainty of the evidence for each pairwise comparison of interventions (14). The risk of bias of comparing each antithrombotic strategy to SAPT in each clinical outcome was visualized as a bar chart (15).

## 3. Results

**Figure 1:**
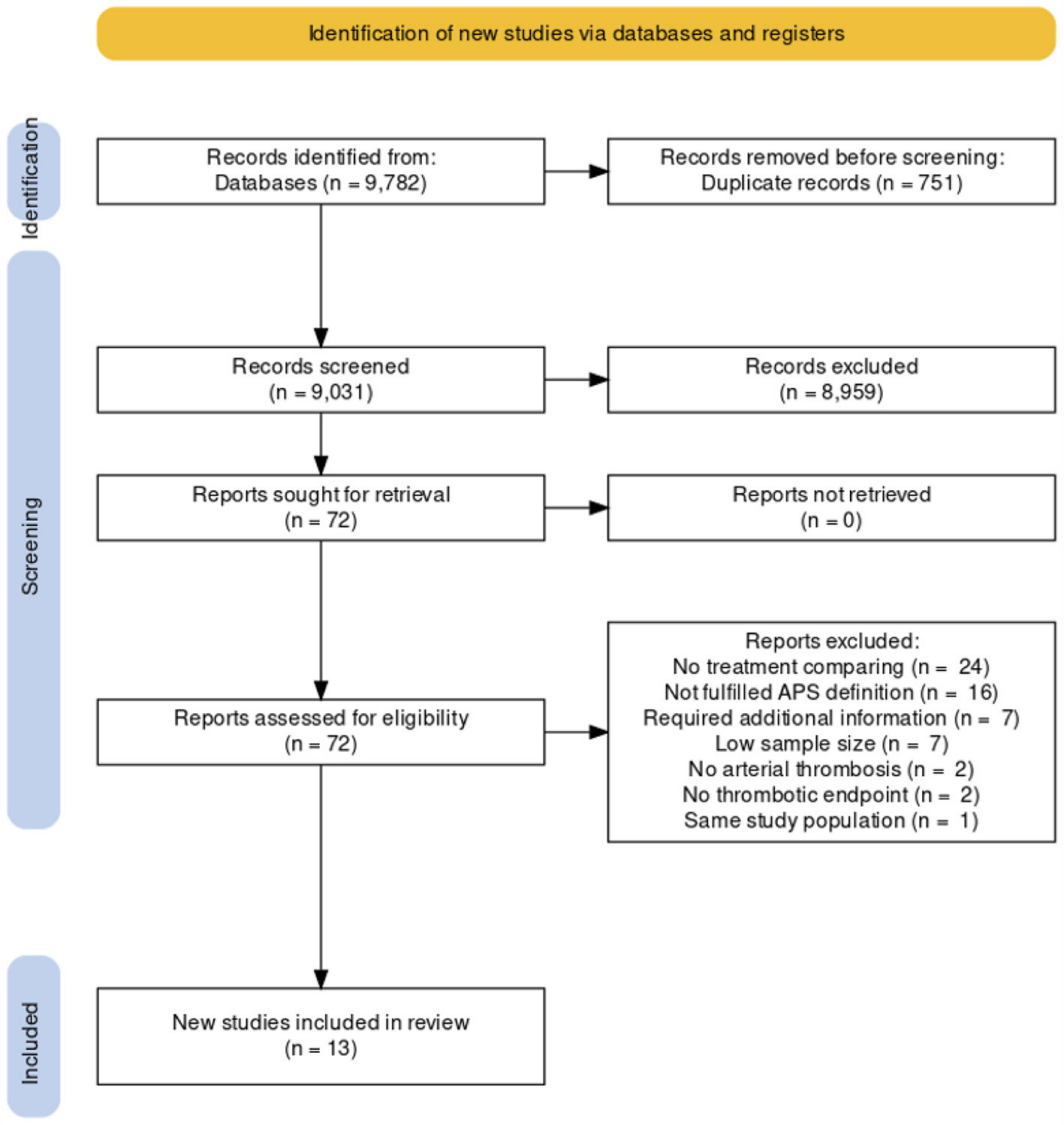
Study screening and selection flow according to PRISMA guidelines.

After excluding duplicated results, the literature search yielded a total of 9,031 studies. After title and abstract screening, 8,959 studies were excluded. A total of 72 studies were included in the full-text reviews. Eight studies required additional information to clarify data, and the corresponding authors of those studies were contacted to request unpublished data (3, 16–22). One of the contacted corresponding authors has responded to our request and provided unpublished data specifically for this analysis (16). Finally, there were 13 studies (six randomized and seven non-randomized studies) included in this NMA, comprising 719 participants (16, 23–34). A diagram summarizing the flow of study selection is shown in Figure 1 (35).

The included studies were published between 2003 and 2022. The number of included participants in this NMA from each study ranged from 17 to 139. There were six randomized studies included in this NMA which involved 238 participants. The initial arterial events were mainly strokes, and five studies included only stroke patients (23, 26, 30, 33, 34). The mean age of participants ranged from 34 to 51 years. The study characteristics and results are summarized in Table 1.

**Table 1:** Summary of included studies in this network meta-analysis.

**Study**	**Study design**	**APS definition**	**Initial arterial event**	**Primary APS**	**Age at include (years)**	**Triple positive APS**	**Number of participants (included in NMA)**	**Intervention**	**Recurrent thrombotic events**	**Major bleeding definition**	**Duration of follow-up (years)**
Crowther et al. (24)	Randomized, double-blinded, multicenter	Sydney criteria	Any arterial thrombosis	86%^*^	Mean (Range): 43 (20–80)^*^	No data	27	Warfarin (INR 3.0–4.0) (*n =* 14)	3 (1A + 2V)	Not defined	Mean 2.7
					Mean (Range): 41 (21–81)^*^			Warfarin (INR 2.0–3.0) (*n =* 13)	1 (1A)		
Yamazaki and Maekawa (33)	Randomized	No data	Stroke	No data	No data	No data	60	Aspirin (*n =* 20)	3 (3A)	Not reported	3
								Aspirin + cilostazol (*n =* 20)	0		
								Aspirin + warfarin (INR 2.0–2.5) (*n =* 20)	0		
Okuma et al. (34)	Randomized, double-blinded, single center (Japan)	Sapporo criteria	Stroke	65%	Mean 48	No data	20	Aspirin (*n =* 11)	8 (8A)	Not defined	Mean ± SD: 3.9 ± 2.0
								Antiplatelet + warfarin (INR 2.0–3.0) (*n =* 9)	2 (2A)		
Arauz et al. (23)	Retrospective, single center (Mexico)	Sapporo criteria	Stroke	100%	Mean ± SD: 33.8 ± 8.9	No data	92	Aspirin (*n =* 38)	0	Not defined	Median (Range): 4.5 (1–20)
								Warfarin (INR 2.5) (*n =* 54)	8 (8A)		
Jackson et al. (25)	Retrospective, multicenter (International)	Sydney criteria	Any arterial thrombosis	65.5%	Median (Range): 43 (5–84)	36%	139	Antiplatelet (aspirin or clopidogrel) (*n =* 38)	16 (A + V)	Not reported	Median 4.24
								Warfarin (INR 2.0–3.0) (*n =* 43)	9 (A + V)		
								Antiplatelet + Warfarin (*n =* 58)	4 (A + V)		
Pyo et al. (30)	Retrospective, single center (Korea)	Sydney criteria	Stroke	78.3%	Mean ± SD: 44.0 ± 13.0	17.2%	46	Antiplatelet (aspirin or clopidogrel) (*n =* 12)	6 (A + V)	Not reported	Mean ± SD: 5.0 ± 4.4
								Aspirin + clopidogrel (*n =* 10)			
								Warfarin (mean INR 2.0) (*n =* 24)	8 (A + V)		
Ohnishi et al. (27)	Retrospective, single center (Japan)	Sydney criteria	Any arterial thrombosis (stroke 90%)	41.1%	Median (IQR): 45 (31–53)	No data	90	Warfarin (INR 1.5–2.5) (*n =* 13)	11 (10A + 1V)	Defined as required hospitalization and/or blood transfusion	Median (IQR): 8 (5–13)
								Antiplatelet (Aspirin (*n =* 34), clopidogrel (*n =* 2), cilostazol (*n =* 4), others (*n =* 1)) (*n =* 41)	18 (16A + 2V)		
								Warfarin + antiplatelet [aspirin (*n =* 18), cilostazol (*n =* 1), others (*n =* 2)] (*n =* 21)	8 (7A + 1V)		
								Dual antiplatelet therapy [aspirin (*n =* 12), clopidogrel (*n =* 10), cilostazol (*n =* 5), others (*n =* 3)] (*n =* 15)	3 (2A + 1V)		
Pengo et al. (29)	Randomized, open-label, multicenter (Italy)	Sydney criteria	Any arterial thrombosis (stroke 64%)	59%^*^	Mean ± SD: 46.5 ± 10.2^*^	100%	43	Rivaroxaban (*n =* 21)	4 (4A)	ISTH definition	Mean 1.6
					Mean ± SD: 46.1 ± 13.2^*^			Warfarin (INR 2.0–3.0) (*n =* 22)	0		
Malec et al. (26)	Prospective cohort, single center (Poland)	Sydney criteria	Stroke	No data	Mean ± SD: 44 ± 11^*^	26.1%^*^	42	DOACs [Apixaban (*n =* 16), Rivaroxaban (*n =* 5), Dabigatran (*n =* 1)] (*n =* 22)	3 (2A + 1V)	ISTH definition	Median (IQR): 4.3 (3.6–5.3)
					Mean ± SD: 45 ± 13^*^			Warfarin (INR 2.0–3.0) (*n =* 20)	4 (2A + 2V)		
Ordi-Ros et al. (28)	Randomized, open-label, multicenter (Spain)	Sydney criteria	Any arterial thrombosis	69.5%^*^	Median (IQR): 47 (40–55)^*^	57.5%^*^	71	Rivaroxaban (*n =* 37)	7 (7A + 1V)	ISTH definition	Mean (range): 3.0 (1.5–3.0)
					Median (IQR): 51 (38–63)^*^			Warfarin (INR 2.0–3.0 or 2.5–3.5 in recurrent thrombosis) (*n =* 34)	3 (2A + 1V)		
Sato et al. (16)	Retrospective, single center (Japan)	Sydney criteria	Any arterial thrombosis	51%^*^	Mean ± SD: 42.8 ± 16.3^*^	37%^*^	34	DOACs [Rivaroxaban (*n =* 3), Edoxaban (*n =* 5)] (*n =* 8)	3 (2A + 1V)	Defined as required hospitalization and/or blood transfusion	5
								Warfarin (INR 2.0–3.0) (*n =* 26)	8 (7A + 1V)		
Franke et al. (31)	Retrospective	Sydney criteria	Any arterial thrombosis	No data	Median (range): 55 (21–81)^*^	3.5%^*^	38	DOACs (mostly rivaroxaban) (*n =* 21)	1 (1A)	Not reported	Median (range): 1.3 (0.1–5.1)
					Median (range): 51 (21–76)^*^			Warfarin (INR 2.0–3.0) (*n =* 17)	0		Median (range): 2.7 (0.3–7.6)
Woller et al. (32)	Randomized, open-label, multicenter (US)	Sydney criteria	Any arterial thrombosis (stroke 71%)	64.6^*^	Mean ± SD: 46.0 ± 11.5^*^	29.2%^*^	17	Apixaban (*n =* 6)	4 (4A)	ISTH definition	1
					Mean ± SD: 48.5 ± 14.4^*^			Warfarin (INR 2–3) (*n =* 11)	0		

A, arterial; APS, antiphospholipid syndrome; V, venous. ^*^No separate data specifically for arterial thrombosis.

### 3.1. Risk-of-bias assessment

A summary of risk of bias within studies for randomized studies is shown in Supplementary Figure 1. Four randomized studies were judged as low risk of overall bias (24, 28, 29, 32). Risk of bias in the randomization process, deviations from the intended interventions, missing outcome data, measurement of the outcome, and selection of the reported result were judged as low risk in these four studies (24, 28, 29, 32). Two randomized studies were judged as having some concern for the risk of bias (33, 34). The summary of risk of bias for non-randomized studies is shown in Supplementary Figure 2. All non-randomized studies were judged to be a moderate risk for the overall risk of bias (16, 23, 25–27, 30, 31). The risk of bias in comparing each antithrombotic strategy to SAPT for recurrent thrombosis is represented as a bar chart, and the grading of evidence is reported in Supplementary Figure 3 and Supplementary Tables 1–5.

### 3.2. Recurrent overall thrombosis

**Figure 2:**
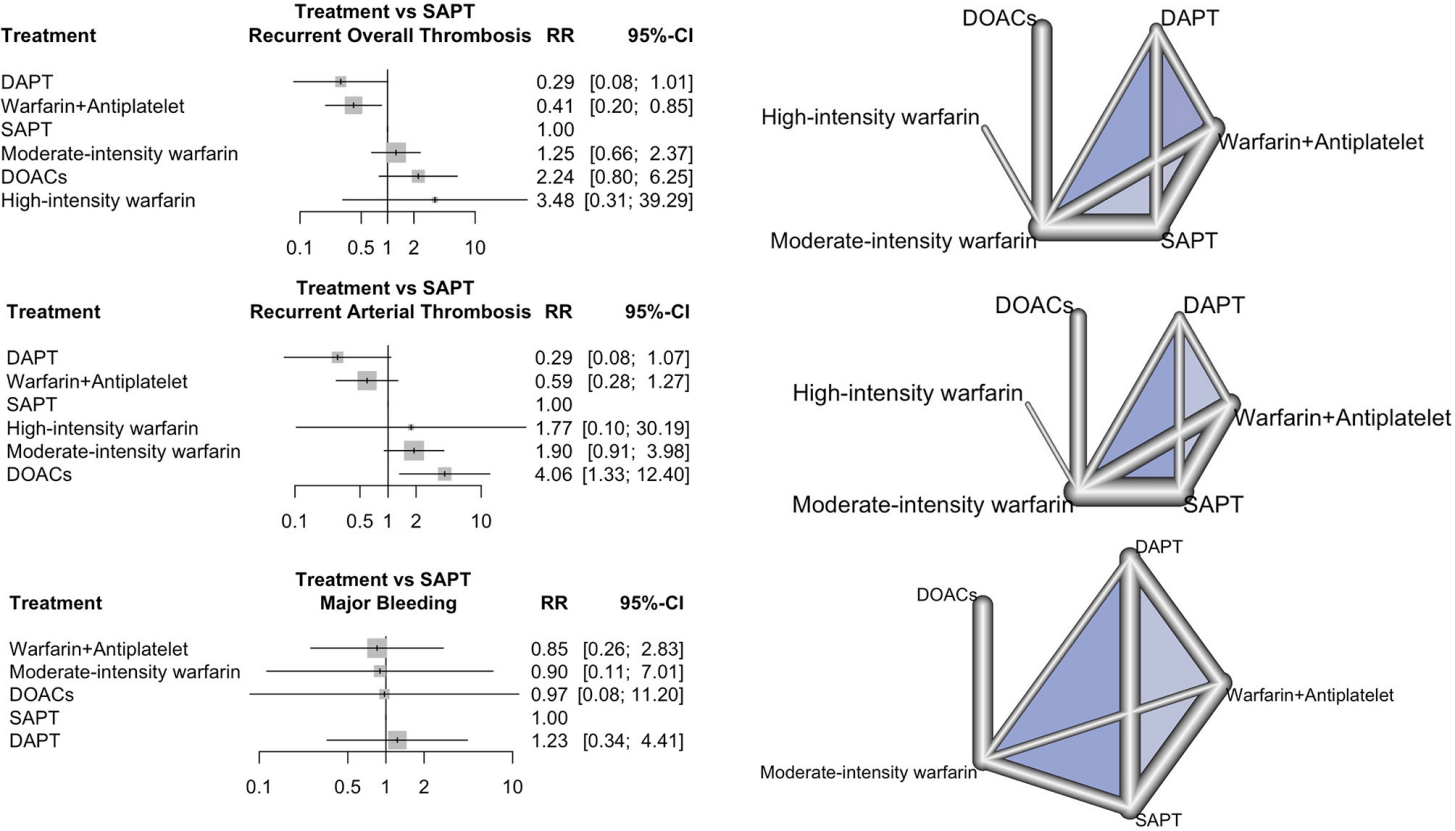
Forest plot and network geometry. The left column shows the forest plot of risk ratio (rr) with a 95% confidence interval (95% CI) of recurrent thrombosis, recurrent arterial thrombosis, and major bleeding outcome comparing each antithrombotic strategy with SAPT as a reference. The right column shows the corresponding network geometry of each outcome (DAPT, dual antiplatelet therapy; DOACs, direct oral anticoagulants; NRCT, non-randomized study; RCT, randomized study; SAPT, single antiplatelet therapy).

Recurrent overall thrombosis was reported in 13 studies, involving 145 (20.2%) participants. The forest plot of the recurrent overall thrombotic event comparing each antithrombotic strategy to SAPT and network geometry are shown in Figure 2. Compared to SAPT, NMA revealed that combined warfarin and antiplatelet were associated with a lower risk of recurrent overall thrombosis with an RR of 0.41 (95% CI 0.20 to 0.85). There was no significant difference in recurrent thrombosis in DAPT, moderated-intensity warfarin, high-intensity warfarin, and DOACs groups compared to SAPT.

DAPT and a combination of warfarin and antiplatelet had the best performance in the prevention of recurrent thrombosis (P-score = 0.9236 and 0.8496, respectively), as shown in Supplementary Table 1. In this NMA, I^2^ was 40.5% (95% CI 0.0% to 69.1%), which reflected moderate inconsistency. There was no heterogeneity between direct and indirect comparisons in this network (*p* = 0.2163 and 0.0648, respectively). The forest plot of the net-splitting method and net heat plot revealed the consistency between direct and indirect evidence (Supplementary Figure 5). Egger's regression test supported no publication bias (*p* = 0.7442). A comparison-adjusted funnel plot is shown in Supplementary Figure 6. The risk of bias for each antithrombotic strategy compared to SAPT, treatment ranking, and grade of evidence for recurrent overall thrombosis are represented in Supplementary Figure 3 and Supplementary Table 1.

### 3.3.Recurrent arterial thrombosis

In total, 11 studies reported 89 recurrent arterial thrombotic events (16.7%) from 534 participants. The forest plot of the recurrent arterial thrombosis comparing each antithrombotic strategy to SAPT and network geometry are shown in Figure 2. Treatment with DAPT and combined warfarin and antiplatelet were associated with lowered risks of recurrent arterial thrombosis with an RR of 0.29 (95% CI 0.08 to 1.07) and an RR of 0.59 (95% CI 0.28 to 1.27) compared to SAPT, respectively. Treatment with DOACs, in contrast, significantly increased the risk of recurrent arterial thrombosis compared to SAPT with an RR of 4.06 (95% CI 1.33 to 12.40). DAPT had the best effective performance as regards the prevention of recurrent arterial thrombosis (P-score = 0.9381), followed by combined warfarin and antiplatelet therapy (P-score = 0.7658), respectively. The NMA of recurrent arterial thrombosis revealed that I^2^ heterogeneity was 11% (95% CI 0.0% to 51.6%), which reflected low heterogeneity. There was no inconsistency between direct and indirect comparisons in this network (*p* = 0.35 and 0.34, respectively). The risk of bias for each antithrombotic strategy compared to SAPT, treatment ranking, and grade of evidence for recurrent arterial thrombosis are shown in Supplementary Figure 3 and Supplementary Table 2.

### 3.4. Venous thrombosis

Venous thrombosis was reported in eight studies (16, 24, 26–29, 31, 32). The forest plot of the venous thrombosis for each antithrombotic strategy compared to SAPT and network geometry are shown in Supplementary Figures 7A, B. There was no significant difference in venous thrombosis among any antithrombotic strategies as compared to SAPT. There was low heterogeneity in the NMA with an I^2^ heterogeneity of 0% (95% CI 0.0% to 2.7%). There was no inconsistency between direct and indirect comparisons (*p* = 0.9346 and 0.5898, respectively). The risk of bias, treatment ranking, and grade of evidence for venous thrombosis are shown in Supplementary Figure 3 and Supplementary Table 3.

### 3.5. Major bleeding

Major bleeding was reported in seven studies (16, 23, 27, 29, 32–34). The forest plot of the risk of major bleeding for each antithrombotic strategy compared to SAPT and network geometry are shown in Figure 2. There was no significant difference in the risk of major bleeding among any antithrombotic strategies as compared to SAPT. There was low heterogeneity in the NMA with an I^2^ heterogeneity of 0.0% (95% CI 0.0% to 21.0%). There was no inconsistency between direct and indirect comparisons (*p* = 0.7885 and 0.7830, respectively). The risk of bias, treatment ranking, and grade of evidence for major bleeding are shown in Supplementary Figure 3 and Supplementary Table 4.

### 3.6. All-cause mortality

All-cause mortality was reported in seven studies (16, 23, 24, 27, 29, 32, 33). Among the participants included in the analysis, a total of 18 individuals died during the course of the study. Four events were related to thrombosis recurrence, and three were bleeding-related events (23, 27, 29). Nine events were from other causes that were not related to antithrombotic treatments and APS (27). Two events were not specified (16). The forest plot of the all-cause mortality for each antithrombotic strategy compared to SAPT and network geometry are shown in Supplementary Figures 7C, D. There was no significant difference in the all-cause mortality among any antithrombotic strategies as compared to SAPT. There was low heterogeneity in the NMA with an I^2^ heterogeneity of 0.0% (95% CI 0.0% to 0.0%). There was no inconsistency between direct and indirect comparisons (*p* = 0.7545 and 0.9049, respectively). The risk of bias, treatment ranking, and grade of evidence for all-cause mortality are shown in Supplementary Figure 3 and Supplementary Table 5.

## 4. Discussion

Our NMA findings suggest that the use of DAPT and combined warfarin and antiplatelet therapy is associated with a lower risk of recurrent overall thrombosis as compared to SAPT in patients diagnosed with APS who have previously experienced arterial thrombosis.

In current clinical practice, the recent EULAR guidelines recommend the consideration of moderate-intensity warfarin, high-intensity warfarin, and, in certain cases, moderated-intensity warfarin in combination with aspirin for secondary thromboprophylaxis in APS patients with arterial thrombosis (5). For stroke patients with confirmed APS, warfarin is recommended over antiplatelet therapy (36). These recommendations are based on supportive evidence from a previous small observational study that included aPL-positive stroke patients without persistently elevated aPL (37).

A combination of warfarin and antiplatelet therapy was recommended as an optional treatment for APS patients with arterial thrombosis based on two studies, namely a small randomized study and a non-randomized study (20, 34). A previous small retrospective study in APS patients reported no recurrent arterial thrombosis in patients who were treated with combined warfarin and aspirin. However, this study included APS patients with both arterial or venous thrombosis, and the total patient years of follow-up were relatively low. Therefore, it could affect the statistical power and generalizability of the results (20). Another randomized study enrolled 20 APS patients with ischemic stroke and reported that a combination of warfarin and antiplatelet therapy significantly lowered the risk of recurrent stroke compared to SAPT (34).

However, it is important to note that the study included only a small number of participants. Moreover, the previous literature on the subject lacks robust evidence regarding the impact of combined antiplatelet and anticoagulant therapy in APS patients specifically presenting with arterial thrombosis. This limitation arises from the small study population and the inclusion of data from patients with both venous and arterial thrombotic events.

Our NMA exclusively included APS patients with arterial thrombosis treated with combined warfarin and antiplatelet therapy from two randomized and two non-randomized studies (25, 27, 33, 34). The results demonstrated the effectiveness of combined warfarin and antiplatelet therapy in preventing recurrent thrombosis, which can specifically be applied to this population.

This meta-analysis revealed that DAPT lowered the risk of recurrent overall and arterial thrombosis in APS patients with arterial thrombosis, although the effect was not statistically significant. DAPT demonstrated the best performance in preventing recurrent overall and arterial thrombosis in this NMA. Aspirin combined with dipyridamole is currently used for secondary thromboprophylaxis in stroke or TIA patients (6). Previous studies, such as the Second European Stroke Prevention Study (ESPS-2) and European/Australasian Stroke Prevention in Reversible Ischemia Trial (ESPRIT), have shown that combining aspirin and dipyridamole significantly reduces the risk of recurrent stroke in stroke patients compared to aspirin alone (38, 39). Despite demonstrating effectiveness in thromboprophylaxis, the evidence of DAPT's effectiveness in secondary thromboprophylaxis in APS is still uncertain. In this NMA, only two studies contributed data exclusively from DAPT in APS patients with arterial thrombosis, including a total of 35 participants. Therefore, further studies are necessary to determine the potential of DAPT as an antithrombotic strategy for thromboprophylaxis in this clinical setting.

The NMA conducted in this study did not demonstrate the superior efficacy of moderate-intensity warfarin over SAPT for the prevention of recurrent thrombosis. One possible explanation for this finding is that patients who received warfarin had subtherapeutic INR levels. For instance, Ohnishi et al. reported a median INR (range) of 2.17 (1.75 to 2.39) in patients with recurrent thrombosis who received warfarin, while Arauz et al. found that all thrombotic events in the warfarin group were associated with an INR level of <2.0 (23, 27). Therefore, to better understand the efficacy of antithrombotic warfarin, additional data on the time in the therapeutic range (TTR) are necessary.

Our study found that APS patients who were treated with DOACs following an arterial thrombotic event had a higher risk of recurrent arterial thrombosis, consistent with previous studies. An international patient-level data meta-analysis, comprising 447 APS patients treated with DOACs across 47 studies, reported a recurrent thrombotic rate as high as 16.0% (40). Similarly, another systematic review and meta-analysis, including 728 APS patients treated with DOACs, showed a 13.9% recurrent thrombosis rate during DOACs treatment, with a majority of patients presenting triple antiphospholipid antibody positivity (48.3%) (41). Based on this evidence, DOAC should be avoided in cases of arterial thrombotic APS.

This study revealed that major bleeding was comparable among the different antithrombotic treatments, which is consistent with other reviews that have compared various antithrombotic strategies (42, 43). However, a limitation of our study is that we were unable to extract data on major bleeding outcomes for APS patients with arterial thrombosis separately from those with venous thrombosis in many of the included studies, and therefore, these data were not included in our network analysis. Another limitation that needs to be considered in interpreting the results of our study is the different definitions of major bleeding used in each study.

The strength of this study is that we exclusively included patients with arterial thrombosis in the analysis. Moreover, we only included studies with persistent aPL elevation, as specified in APS diagnostic criteria, in our network to apply our results to the APS population. Second, due to limited evidence comparing the efficacy of each antithrombotic strategy in this setting and to compare the effects of multiple treatment regimens, we performed NMA, which was appropriate and effective in assessing the efficacy of each antithrombotic strategy. The NMA added an advantage over conventional meta-analysis by integrating the analysis from multiple direct and indirect comparisons, which is more suitable for analyzing outcomes in this setting. Third, we reported all important outcomes, including recurrent overall thrombosis, recurrent arterial thrombosis, and major bleeding. Finally, we assessed the potential risk of bias from both included studies and the method of NMA using several measurements.

However, there are some limitations to this study. First, there was heterogeneity in the quality of the included studies. To address this concern, we performed a publication bias assessment for all outcomes and found no evidence of potential bias. The major concern for NMA applications was network inconsistency, which we assessed using various methods mentioned above, and we found no inconsistency within our network. Furthermore, we assessed the risk of bias for each treatment and compared the level of certainty of our results. Second, there was variation in follow-up duration among the included studies. The differences in the number of events observed across studies could be attributed to varying lengths of follow-up periods. Third, we were not able to perform an analysis based on the vascular bed involved at presentation (MI, stroke, or peripheral arterial disease). Therefore, the results represent patients who presented with an arterial thrombotic event as a whole group. Finally, we were not able to disaggregate patients based on the type of antiplatelet therapy used.

## 5. Conclusion

To the best of our knowledge, this is the first NMA to evaluate the efficacy and safety of multiple antithrombotic strategies in patients with APS and arterial thrombosis. Our NMA demonstrated that combined warfarin and antiplatelet therapy is an effective regimen for secondary thromboprophylaxis in this population. DAPT may also be a potential antithrombotic strategy in this setting; however, the study population is still limited. The use of DOACs should be avoided in this setting due to an increased risk of recurrent arterial thrombosis. Further high-quality randomized studies or large registries are needed to determine the optimal treatment for this population.

## Data availability statement

The original contributions presented in the study are included in the article/Supplementary material, further inquiries can be directed to the corresponding author.

## Author contributions

TA and CC-A contributed to the concept, created search terms, prepared the original manuscript, and performed the statistical analysis. TA and AA performed a search selection, extracted data, and graded the risk of bias. CC-A revised the manuscript. All authors critically reviewed the study results and a final manuscript.
