# Supplementary material for: Antithrombotic therapy in antiphospholipid syndrome with arterial thrombosis: a systematic review and network meta-analysis
Source: Front Med (Lausanne). 2023 Jun 15;10:1196800. doi: 10.3389/fmed.2023.1196800 (PMC10311019; doi:10.3389/fmed.2023.1196800)
Supplement: Supplementary file 1 [file Table_1.DOCX]

***Supplementary material***

**Antithrombotic therapy in Antiphospholipid syndrome with arterial thrombosis: A systematic review and network meta-analysis**

Tanawat Attachaipanich^1^, Aimpat Aungsusiripong^2^, Pokpong Piriyakhuntorn^3^, Sasinee Hantrakool^3^, Ekarat Rattarittamrong^3^, Thanawat Rattanathammethee^3^, Adisak Tantiworawit^3^, Lalita Norrasethada^3^, Chatree Chai-Adisaksopha^3*^

*Correspondence: Chatree Chai-Adisaksopha: [Chatree.chai@cmu.ac.th](mailto:Chatree.chai@cmu.ac.th)

1 Supplementary Data

**1.1 Search strategies**

**1.1.1 Search strategy for OVID MEDLINE** (1845 results)

1. exp Antiphospholipid Syndrome/

2. exp Antibodies, Antiphospholipid/

3. exp Antibodies, Anticardiolipin/

4. exp beta 2-Glycoprotein I/

5. exp Lupus Coagulation Inhibitor/

6. 1 or 2 or 3 or 4 or 5
7. exp Thrombosis/

8. exp Stroke/

9. exp Cerebrovascular Disorders/

10. exp Acute Coronary Syndrome/

11. exp Coronary Artery Disease/

12. exp Myocardial Infarction/

13. exp Venous Thrombosis/

14. exp Thromboembolism/

15. exp Pulmonary Embolism/

16. exp Peripheral Artery Disease/

17. 7 or 8 or 9 or 10 or 11 or 12 or 13 or 14 or 15 or 16

18. exp Aspirin/

19. exp Anticoagulants/

20. exp Warfarin/

21. exp Heparin/

22. exp Factor Xa Inhibitors/

23. exp Rivaroxaban/

24. apixaban.mp.

25. edoxaban.mp.

26. exp Dabigatran/

27. 18 or 19 or 20 or 21 or 22 or 23 or 24 or 25 or 26

28. 6 and 17 and 27

**1.1.2 Search strategy for EMBASE** (5144 results)

1. ‘antiphospholipid syndrome’

2. ‘phospholipid antibody’

3. ‘cardiolipin antibody’

4. ‘beta2 glycoprotein 1’

5. ‘lupus anticoagulant’

6. #1 OR #2 OR #3 OR #4 OR #5
7. ‘thrombosis’

8. ‘cerebrovascular accident’

9. ‘cerebrovascular disease’

10. ‘acute coronary syndrome’

11. ‘coronary artery disease’

12. ‘heart infarction’

13. ‘vein thrombosis’

14. ‘thromboembolism’

15. ‘lung embolism’

16. ‘peripheral occlusive artery disease’

17. #7 OR #8 OR #9 OR #10 OR #11 OR #12 OR #13 OR #14 OR #15 OR #16

18. ‘acetylsalicylic acid’

19. ‘anticoagulant agent’

20. ‘warfarin’

21. ‘heparin derivative’

22. ‘blood clotting factor 10a inhibitor’

23. ‘rivaroxaban’

24. ‘apixaban’

25. ‘edoxaban’

26. ‘dabigatran’

27. #18 OR #19 OR #20 OR #21 OR #22 OR #23 OR #24 OR #25 OR #26

28. #6 AND #17 AND #27

29. #6 AND #17 AND #27 AND ([article]/lim OR [article in press]/lim OR [conference abstract]/lim OR [conference paper]/lim OR [conference review]/lim OR [data papers]/lim OR [letter]/lim OR [preprint]/lim) AND [humans]/lim AND [embase]/lim

**1.1.3 Search strategy for Web of Science (2747 results)**

#1 TS=(“antiphospholipid syndrome”) or TS=(“antiphospholipid antibod*”) or TS=(anticardiolipin) or TS=(“lupus anticoagulant”) or TS=(“beta 2-glycoprotein I”)

#2 TS=(“arterial thrombosis”) or TS=(stroke) or TS=(“cerebrovascular disorder”) or TS=(“acute coronary syndrome”) or TS=(“coronary artery disease”) or TS=(“myocardial infarction”) or TS=(Peripheral artery disease) or TS=(“venous thrombosis”) or TS=(“thromboembolism”) or TS=(“pulmonary embolism”) or TS=(“deep vein thrombosis”)

#3 TS=(aspirin) or TS=(anticoagulant*) or TS=(warfarin) or TS=(enoxaparin) or TS=(tinzaparin) or TS=(dalteparin) or TS=(“Factor Xa inhibitor*”) or TS=(rivaroxaban) or TS=(apixaban) or TS=(edoxaban) or TS=(dabigatran)

#4 #1 AND #2 AND #3

**1.1.4 Search strategy for Cochrane Library (46 results)**

#1 MeSH descriptor: [Antiphospholipid Syndrome] explode all trees

#2 MeSH descriptor: [Antibodies, Antiphospholipid] explode all trees

#3 MeSH descriptor: [Lupus Coagulation Inhibitor] explode all trees

#4 MeSH descriptor: [Antibodies, Anticardiolipin] explode all trees

#5 MeSH descriptor: [beta 2-Glycoprotein I] explode all trees

#6 #1 or #2 or #3 or #4 or #5

#7 MeSH descriptor: [Thrombosis] explode all trees

#8 MeSH descriptor: [Coronary Thrombosis] explode all trees

#9 MeSH descriptor: [Coronary Artery Disease] explode all trees

#10 MeSH descriptor: [Acute Coronary Syndrome] explode all trees

#11 MeSH descriptor: [Myocardial Infarction] explode all trees

#12 MeSH descriptor: [Peripheral Arterial Disease] explode all trees

#13 MeSH descriptor: [Stroke] explode all trees

#14 MeSH descriptor: [Venous Thrombosis] explode all trees

#15 MeSH descriptor: [Thromboembolism] explode all trees

#16 MeSH descriptor: [Pulmonary Embolism] explode all trees

#17 #8 or #9 or #10 or #11 or #12 or #13 or #14 or # 15 or #16

#18 #6 and #17

2 Supplementary Figures and Tables

**2.1 Supplementary Figures**

**
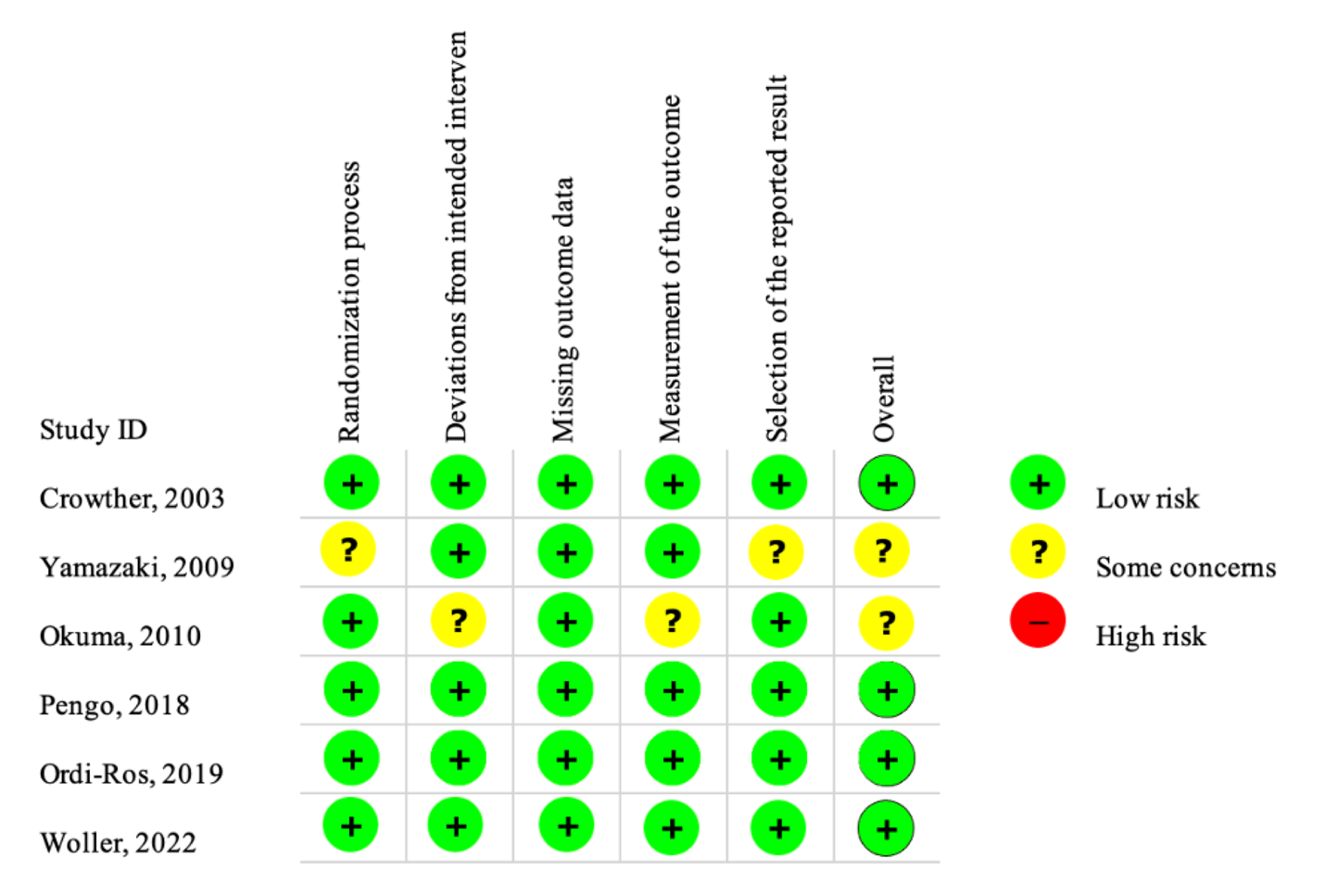
**

**Supplementary Figure 1.** Risk-of-bias for randomized studies. Evaluated using Revised Cochrane risk-of-bias tool for randomized trials (RoB 2).


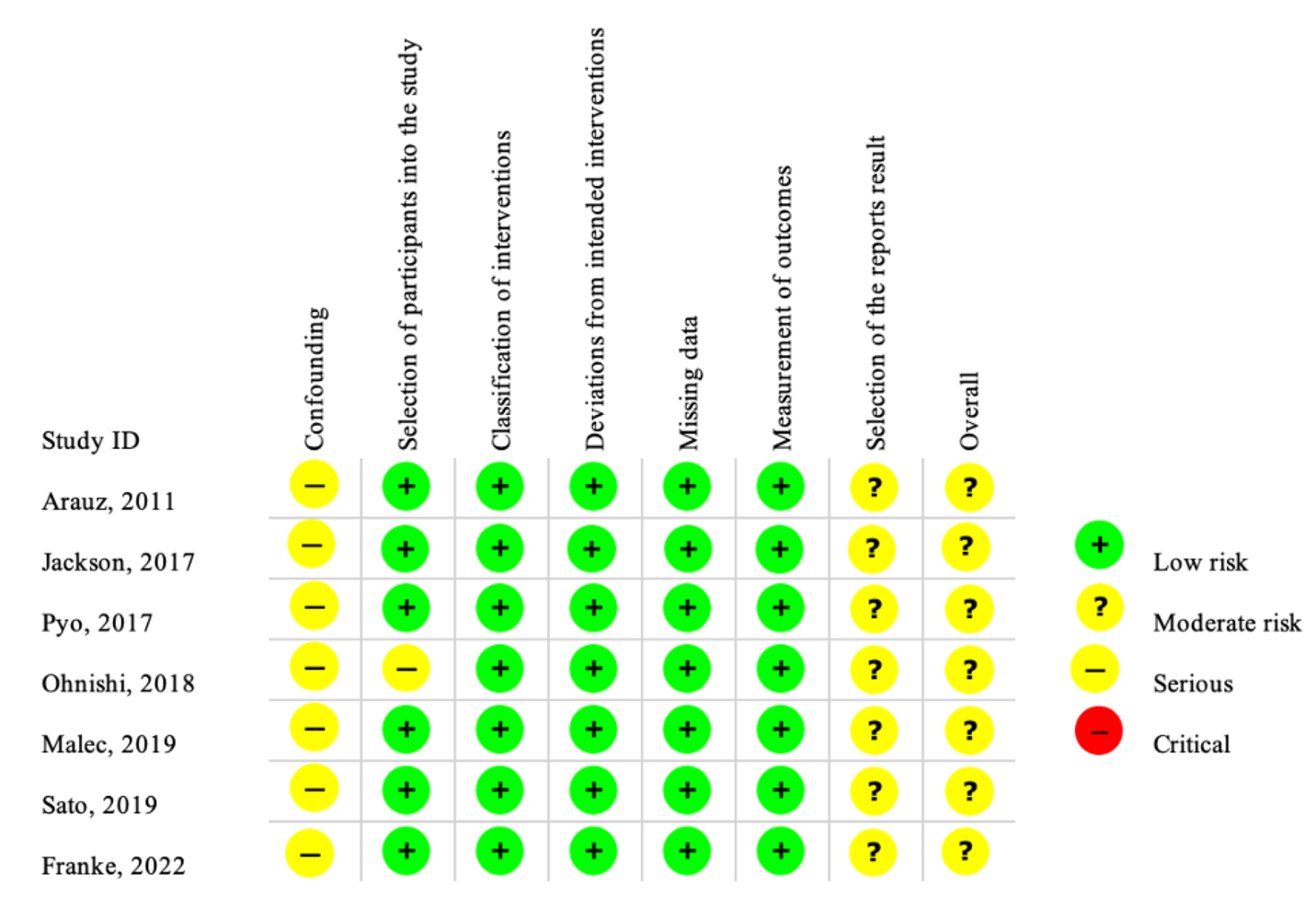


**Supplementary Figure 2.** Risk-of-bias for non-randomized studies. Evaluated using Risk Of Bias In Non-randomized Studies of Interventions (ROBINS-I).


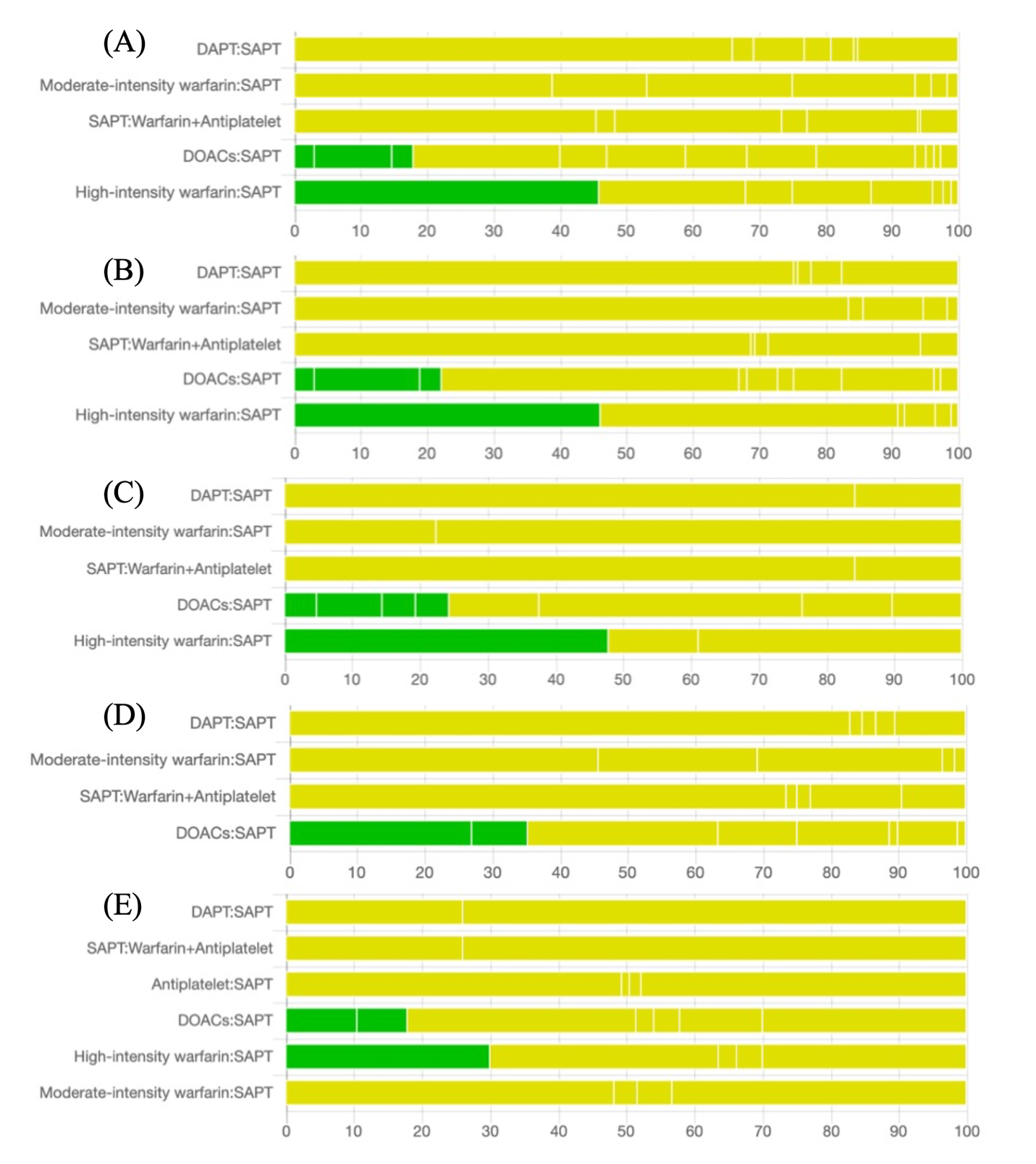


**Supplementary Figure 3.** The bar chart represented risk-of-bias in comparing each antithrombotic strategy compared to SAPT for each outcome. (A) Recurrent thrombosis, (B) Recurrent arterial thrombosis, (C) Venous thrombosis, (D) Major bleeding, and (E) All-cause mortality. Color in the bar chart was green and yellow corresponding with the risk-of-bias as low risk and some concerns respectively. *(abbreviations: DAPT; Dual antiplatelet therapy, DOACs; Direct oral anticoagulants, SAPT; Single antiplatelet therapy)*


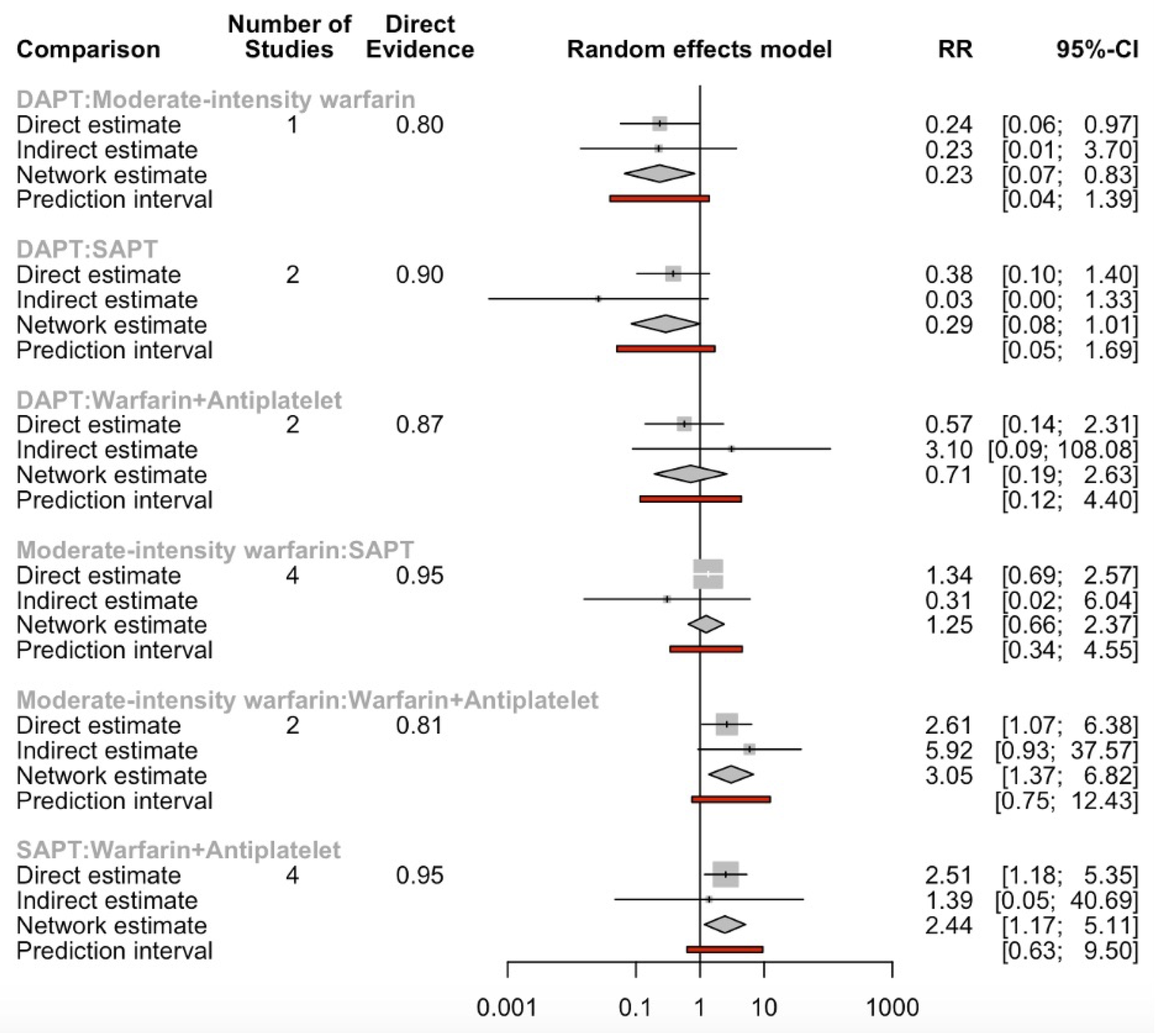


**Supplementary Figure 4.** Net splitting plots of recurrent thrombosis. The result is represented in Risk ratio (RR) and 95% confidence interval (CI). *(abbreviations: DAPT; Dual antiplatelet therapy, SAPT; Single antiplatelet therapy)*

**
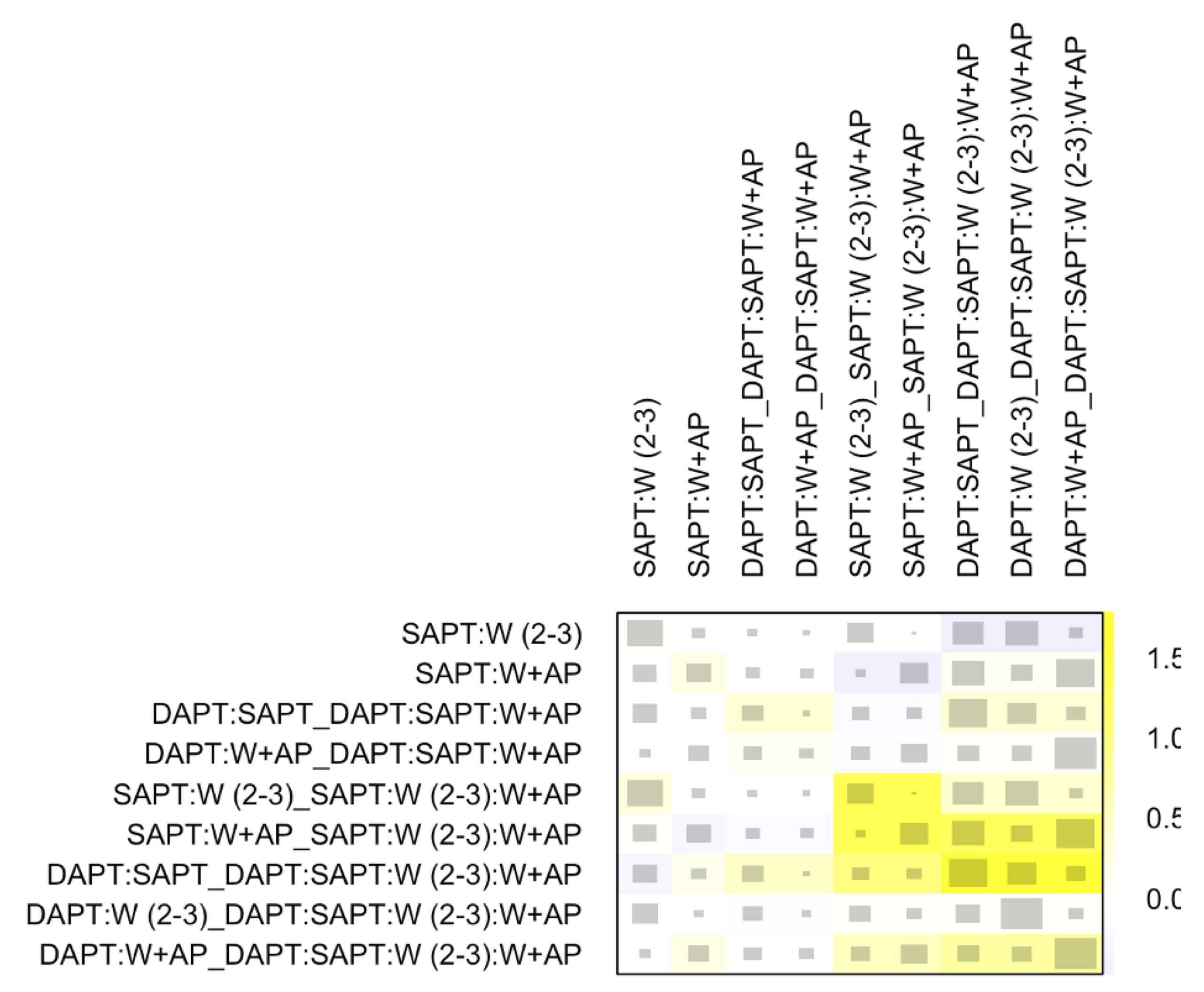
**

**Supplementary Figure 5.** Net heat plot. The size of the grey box represented the importance of a treatment comparison for estimating the network evidence. The color background from blue to red represented the inconsistency of the comparison.  *(abbreviations: DAPT; Dual antiplatelet therapy, SAPT; Single antiplatelet therapy, W (2-3); Moderate-intensity warfarin, W (3-4); High-intensity warfarin, W+AP; Combined warfarin and antiplatelet)*

*
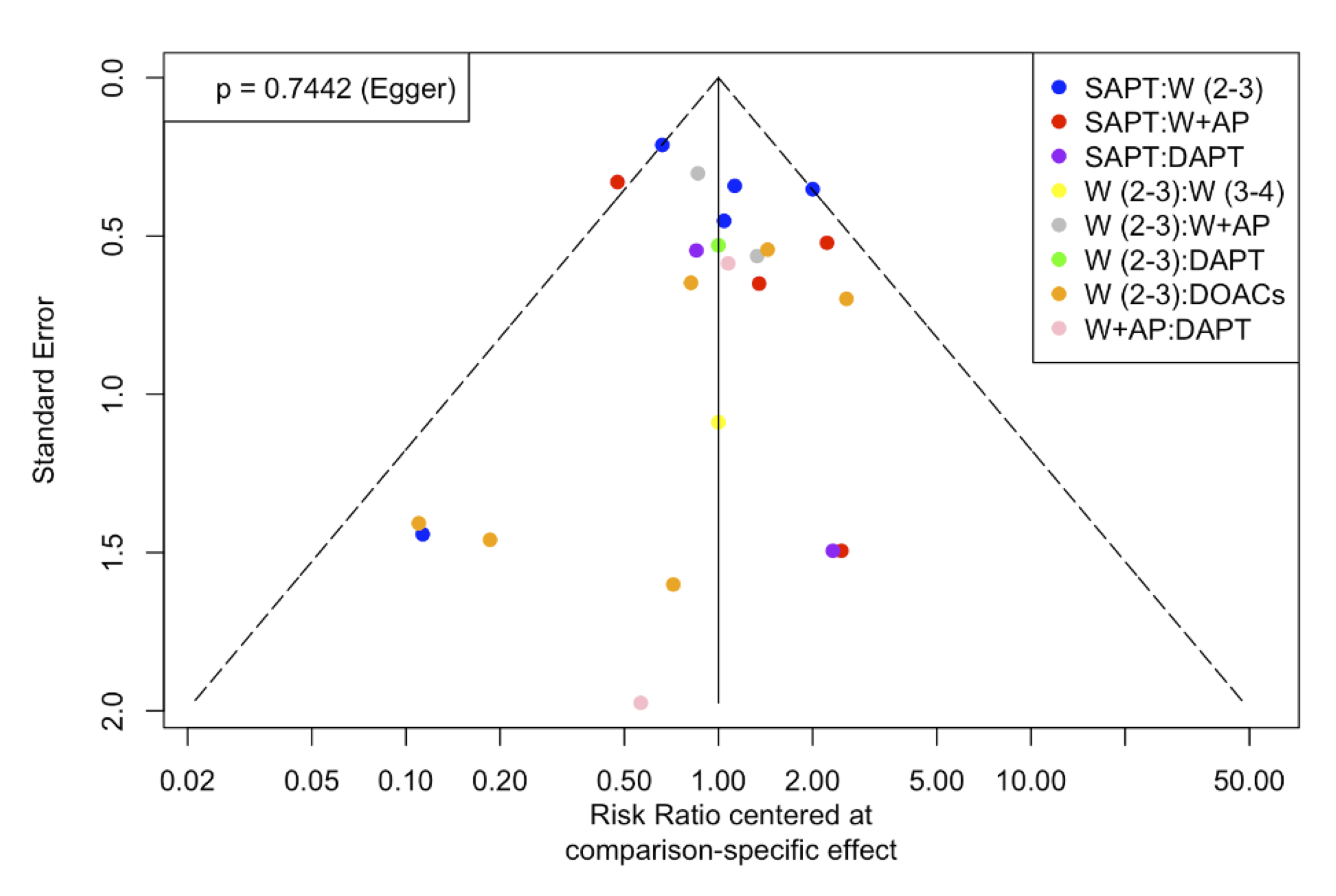
*

**Supplementary Figure 6.** Comparison-adjusted funnel plot. Represented publication bias *(abbreviations: DAPT; Dual antiplatelet therapy, DOACs; Direct oral anticoagulants, SAPT; Single antiplatelet therapy, W (2-3); Moderate-intensity warfarin, W (3-4); High-intensity warfarin, W+AP; Combined warfarin and antiplatelet)*

**
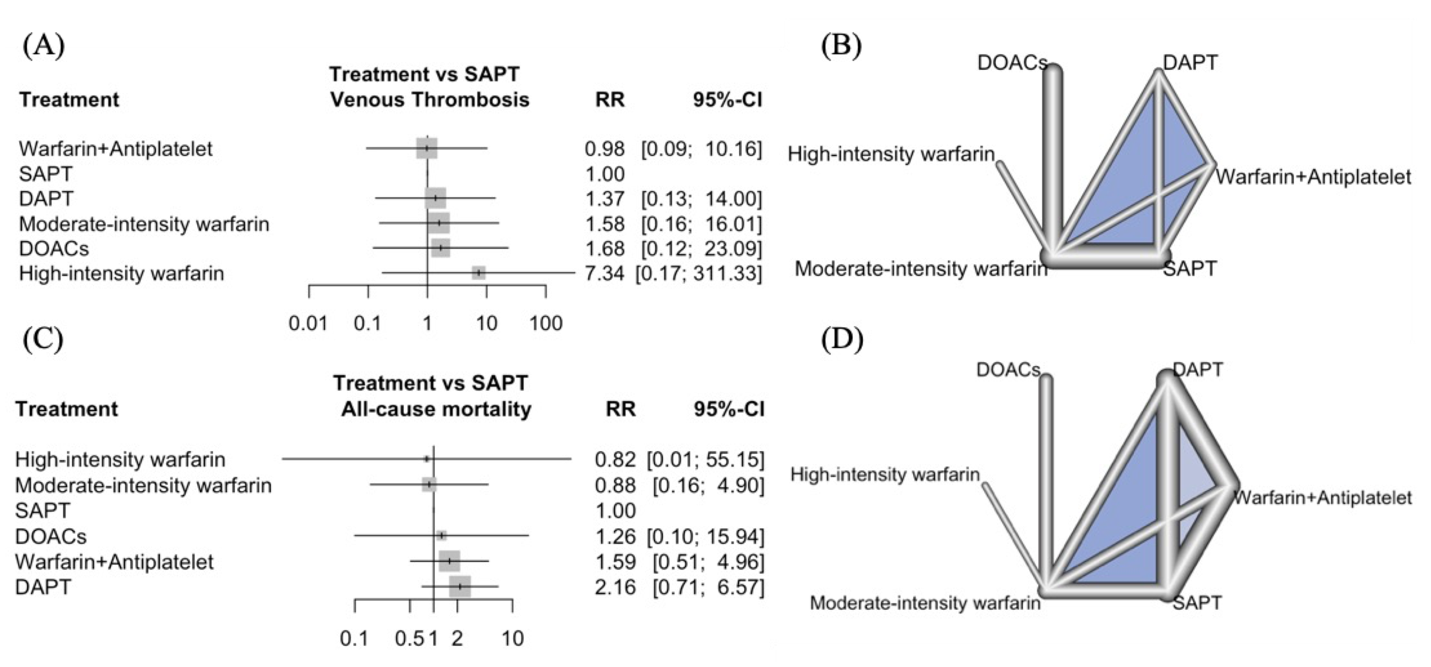
**

**Supplementary Figure 7.** Forest plot and network geometry of venous thrombosis and network geometry. The left column showed the forest plot of Risk ratio (RR) with 95% confidence interval (95%CI) of venous thrombosis and all-cause mortality comparing each antithrombotic strategy with SAPT as a reference. The right column showed the corresponding network geometry of each outcome. Supplementary Figures (A) and (B) demonstrate the forest plot and network geometry of venous thrombosis, and (C) and (D) for All-cause mortality. *(abbreviations: DAPT; Dual antiplatelet therapy, DOACs; Direct oral anticoagulants, SAPT; Single antiplatelet therapy)*

**2.2 Supplementary Tables**

| **Treatment** | **Total study and participants in direct comparison with SAPT** | **Risk ratio (RR) (95% CI) from network estimation** | **Certainty of evidence** | **Ranking (p-score)** |
| --- | --- | --- | --- | --- |
| SAPT | Reference comparator | No estimation | - | Rank 3  (0.5135) |
| DAPT | 1 RCT, 1 NRCT, 96 participants | 0.29 (95% CI 0.08 to 1.01) | very low^1,3^ | Rank 1 (0.9236) |
| Combined warfarin and antiplatelet | 2 RCT, 2 NRCT, 223 participants | 0.41 (95% CI 0.20 to 0.85) | very low^1,2^ | Rank 2  (0.8496) |
| Moderate-intensity warfarin | 4 NRCT, 273 participants | 1.25 (95% CI 0.66 to 2.37) | very low^1,3^ | Rank 4  (0.3986) |
| High-intensity warfarin | No direct comparison | 3.48 (95%CI 0.31 to 39.29) | very low^1,3,4^ | Rank 5  (0.1590) |
| DOACs | No direct comparison | 2.24 (95%CI 0.80 to 6.25) | very low^1,3,4^ | Rank 6  (0.1557) |

1 downgrade by one due to risk of bias, 2 downgrade by one due to inconsistency, 3 downgrade by one due to imprecision, 4 downgrade by one due to indirectness

**Supplementary Table 1.** Summary of findings table for recurrent overall thrombosis. Represented included a total number of included studies and participants of direct comparisons between each antithrombotic strategy compared to SAPT as a reference. Risk ratio (RR) (95%CI), and treatment ranking (p-score) were estimated from the network. The grading of evidence in each antithrombotic regimen compared to SAPT. *(abbreviations: DAPT; Dual antiplatelet therapy, DOACs; Direct oral anticoagulants, NRCT; Non-randomized study, RCT; Randomized study, SAPT; Single antiplatelet therapy)*

| **Treatment** | **Total study and participants in direct comparison with SAPT** | **Risk ratio (RR) (95% CI) from network estimation** | **Certainty of evidence** | **Ranking (p-score)** |
| --- | --- | --- | --- | --- |
| SAPT | Reference comparator | No estimation | - | Rank 3  (0.5450) |
| DAPT | 1 RCT, 1 NRCT, 96 participants | 0.29 (95% CI 0.08 to 1.07) | very low^1,3^ | Rank 1 (0.9381) |
| Combined warfarin and antiplatelet | 2 RCT, 1 NRCT, 183 participants | 0.59 (95% CI 0.28 to 1.27) | very low^1,2^ | Rank 2  (0.7658) |
| High-intensity warfarin | No direct comparison | 1.77 (95%CI 0.10 to 30.19) | very low^1,3,4^ | Rank 4  (0.3868) |
| Moderate-intensity warfarin | 2 NRCT, 146 participants | 1.90 (95% CI 0.91 to 3.98) | very low^1,3^ | Rank 5  (0.2981) |
| DOACs | No direct comparison | 4.06 (95%CI 1.33 to 12.40) | very low^1,3,4^ | Rank 6  (0.0663) |

1 downgrade by one due to risk of bias, 2 downgrade by one due to inconsistency, 3 downgrade by one due to imprecision, 4 downgrade by one due to indirectness

**Supplementary Table 2.** Summary of findings table for recurrent arterial thrombosis. Represented included a total number of included studies and participants of direct comparisons between each antithrombotic strategy compared to SAPT as a reference. Risk ratio (RR) (95%CI), and treatment ranking (p-score) were estimated from the network. The grading of evidence in each antithrombotic regimen compared to SAPT. *(abbreviations: DAPT; Dual antiplatelet therapy, DOACs; Direct oral anticoagulants, NRCT; Non-randomized study, RCT; Randomized study, SAPT; Single antiplatelet therapy)*

| **Treatment** | **Total study and participants in direct comparison with SAPT** | **Risk ratio (RR) (95% CI) from network estimation** | **Certainty of evidence** | **Ranking (p-score)** |
| --- | --- | --- | --- | --- |
| SAPT | Reference comparator | No estimation | - | Rank 1  (0.6495) |
| Combined warfarin and antiplatelet | 1 NRCT, 62 participants | 0.98 (95% CI 0.09 to 10.16) | very low^1,3^ | Rank 2  (0.6443) |
| DAPT | 1 NRCT, 56 participants | 1.37 (95% CI 0.13 to 14.00) | very low^1,3^ | Rank 3 (0.5384) |
| Moderate-intensity warfarin | 2 NRCT, 89 participants | 1.58 (95% CI 0.16 to 16.01) | very low^1,3^ | Rank 4  (0.5115) |
| DOACs | No direct comparison | 1.68 (95%CI 0.12 to 23.09) | very low^1,3,4^ | Rank 5  (0.4868) |
| High-intensity warfarin | No direct comparison | 7.34 (95%CI 0.17 to 311.33) | very low^1,3,4^ | Rank 6  (0.1695) |

1 downgrade by one due to risk of bias, 2 downgrade by one due to inconsistency, 3 downgrade by one due to imprecision, 4 downgrade by one due to indirectness

**Supplementary Table 3.** Summary of findings table for venous thrombosis. Represented included a total number of included studies and participants of direct comparisons between each antithrombotic strategy compared to SAPT as a reference. Risk ratio (RR) (95%CI), and treatment ranking (p-score) were estimated from the network. The grading of evidence in each antithrombotic regimen compared to SAPT. *(abbreviations: DAPT; Dual antiplatelet therapy, DOACs; Direct oral anticoagulants, NRCT; Non-randomized study, RCT; Randomized study, SAPT; Single antiplatelet therapy)*

| **Treatment** | **Total study and participants in direct comparison with SAPT** | **Risk ratio (RR) (95% CI) from network estimation** | **Certainty of evidence** | **Ranking (p-score)** |
| --- | --- | --- | --- | --- |
| SAPT | Reference comparator | No estimation | - | Rank 4  (0.4925) |
| Combined warfarin and antiplatelet | 2 RCT, 1 NRCT, 122 participants | 0.85 (95% CI 0.26 to 2.83) | very low^1,2^ | Rank 1  (0.5879) |
| Moderate-intensity warfarin | 2 NRCT, 146 participants | 0.90 (95% CI 0.11 to 7.01) | very low^1,3^ | Rank 2  (0.5447) |
| DOACs | No direct comparison | 0.97 (95%CI 0.08 to 11.20) | very low^1,3,4^ | Rank 3  (0.4971) |
| DAPT | 1 RCT, 1 NRCT, 96 participants | 1.23 (95% CI 0.34 to 4.41) | very low^1,3^ | Rank 5 (0.3778) |
| High-intensity warfarin | Not involve in network | - | - | - |

1 downgrade by one due to risk of bias, 2 downgrade by one due to inconsistency, 3 downgrade by one due to imprecision, 4 downgrade by one due to indirectness

**Supplementary Table 4.** Summary of findings table for major bleeding. Represented included a total number of included studies and participants of direct comparisons between each antithrombotic strategy compared to SAPT as a reference. Risk ratio (RR) (95%CI), and treatment ranking (p-score) were estimated from the network. The grading of evidence in each antithrombotic regimen compared to SAPT. *(abbreviations: DAPT; Dual antiplatelet therapy, DOACs; Direct oral anticoagulants, NRCT; Non-randomized study, RCT; Randomized study, SAPT; Single antiplatelet therapy)*

| **Treatment** | **Total study and participants in direct comparison with SAPT** | **Risk ratio (RR) (95% CI) from network estimation** | **Certainty of evidence** | **Ranking (p-score)** |
| --- | --- | --- | --- | --- |
| SAPT | Reference comparator | No estimation | - | Rank 2  (0.6348) |
| Moderate-intensity warfarin | 2 NRCT, 146 participants | 0.88 (95% CI 0.16 to 4.90) | very low^1,3^ | Rank 1  (0.6519) |
| High-intensity warfarin | No direct comparison | 0.82 (95%CI 0.01 to 55.15) | very low^1,3,4^ | Rank 3  (0.5849) |
| DOACs | No direct comparison | 1.26 (95%CI 0.10 to 15.94) | very low^1,3,4^ | Rank 4  (0.4860) |
| Combined warfarin and antiplatelet | 1 RCT, 1 NRCT, 102 participants | 1.59 (95% CI 0.51 to 4.96) | very low^1,3^ | Rank 5  (0.3973) |
| DAPT | 1 RCT, 1 NRCT, 96 participants | 2.16 (95% CI 0.71 to 6.57) | very low^1,3^ | Rank 6 (0.2449) |

1 downgrade by one due to risk of bias, 2 downgrade by one due to inconsistency, 3 downgrade by one due to imprecision, 4 downgrade by one due to indirectness

**Supplementary Table 5.** Summary of findings table for all-cause mortality. Represented included a total number of included studies and participants of direct comparisons between each antithrombotic strategy compared to SAPT as a reference. Risk ratio (RR) (95%CI), and treatment ranking (p-score) were estimated from the network. The grading of evidence in each antithrombotic regimen compared to SAPT. *(abbreviations: DAPT; Dual antiplatelet therapy, DOACs; Direct oral anticoagulants, NRCT; Non-randomized study, RCT; Randomized study, SAPT; Single antiplatelet therapy)*
